# Antivenom Efficacy in Neutralizing Histopathological Complications Following *Latrodectus dahli* Envenomation

**Published:** 2017-03-14

**Authors:** Elham Valikhanfard-Zanjani, Abbas Zare-Mirakabadi, Ehsan Zayerzadeh

**Affiliations:** 1 Department of Biology, Faculty of Sciences, Kharazmi University, Tehran, Iran; 2 Department of Venomous Animals and Antivenom Production, Razi Vaccine and Serum Research Institute, Karaj, Iran; 3 Department of Biology, Faculty of Food Industry and Agriculture, Standard Research Institute, Karaj, Iran

**Keywords:** *Latrodectus*, Antivenom, Histopathological complication

## Abstract

**Background:**

Nowadays use of specific antivenin for latrodectism is considered as the most effective treatment in the world. This study was undertaken to investigate the efficacy of specific antivenom against histopathological complications caused by *Latrodectus dahli* venom on liver, heart and kidneys tissues within 72h.

**Methods:**

Two groups were selected, each one contained 6 male New Zealand rabbits weighing 2±0.5kg. The animals were anesthetized with 0.5ml ketamine and 0.5ml xylazine by intramuscular route. The *L. dahli* venom (0.5mg/kg) was injected subcutaneously to both the groups. The second group of rabbits 24h after the venom injection received specific antivenom by intravenous route. Seventy-two hours after the venom and antivenom injections, the rabbits were dissected to obtain heart, liver and kidney tissues. The tissues were stained by hematoxylin and eosin stains and histopathological studies were examined by optical microscope.

**Results:**

In group one, the venom induced myocytolysis, myocarditis, coagulation necrosis in the heart tissue and the liver tissue showed central vein congestion, congested vessels, dilated sinusoids and inflammation. However, no significant histopathological complications were observed in kidney tissues. In the second group, antivenom injection greatly prevented escalation of the complications on foresaid tissues.

**Conclusion:**

*Latrodectus dahli* venom induces histopathological complications on vital organs. Specific antivenom injection, 24h after the venom injection, could protect the tissues from incidence and intensification of histopathological complications. Future studies in human beings should be conducted to assess the protection against the specific-*Latrodectus* antivenin.

## Introduction

Black widow spider envenomation, because of systemic complications, sometimes fatal, has become as a candidate for the most dangerous arachnids in the world (Vetter and Isbister 2007, Rey et al. 2011). Among more than 40 species that are identified in all over the world, only 4 species were reported as inhabitant of Iran which including *Latrodectus tredecimgottatus*, *L. dahli*, *L. geometricus* and *L. pallidus* (Rafinejad et al. 2007, Afshari et al. 2009, Shahi et al. 2011). The *Latrodectus* spp. are predominantly distributed in the North East and the South of the country.

Latrodectism, syndrome caused by *Latrodectus* spp., generally is characterized by pain, muscular weakness, nausea, vomiting, painful abdominal cramping, perspiration and cardiovascular complications (Marzan 1955, Maretic 1983, Prior and Park 2004). The most reports of latrodectism in Iran return to the 1994, so that nearly 190 cases of latrodectism were reported only in the Khorasan Province (Mirshamsi kakhaki 2005, Afshari et al. 2009).

Black widow spider venom contains a neurotropic protein termed α-Latrotoxin which is thought to be responsible for the most clinical effects in human (Nelson et al. 2011, Nentwig and Nentwig 2013). α-LTX exerts its destructive effects through interaction with specific receptors termed neurexin and latrophilin, so that leads to neurotransmitter depletion of both cholinergic and adrenergic terminals. Given that the highest expression of the receptors are in neurons of nervous system, so it seems that nervous system is the primary target of black widow spider venom (Henkel and Sankaranarayanan 1999, Ushkaryov et al. 2008, Silva and Ushkaryov 2010). Duration of latrodectism in untreated cases can be different in ranges, from a few hours to several days (Malley et al. 1999, Vetter and Isbister 2007). In the past, in order to treat symptoms caused by latrodectism, various medications were used, like calcium gluconate and benzodiazepines. The success rates of these drugs are different but all of them were used just to relieve pain and other symptoms caused by envenomation (Allen and Norris 1991, Clark et al. 1992).

Nowadays usage of antibodies and antibody fragments are considered as the most effective treatment in latrodectism. However, there is a substantial controversy about the route and time of black widow spider antivenom injection, so that previously black widow specific antivenom was injected intramuscularly, but according to the published data in recent decades, intramuscular route is not efficient, so intravenous route was proposed. However, determination of antivenom ability to reverse complications of black widow envenomation needs more investigations (Isbister 2002, Brown et al. 2007, Ahmed and Bushra 2008).

So far, most of clinical studies performed on latrodectism, were concerning on the venom effects on organs functions. Considerably less attention has been paid to venom effects on organs tissues. Expressions of the certain alpha latrotoxin receptors on some of the mammalian tissues, makes the direct effects of venom expectable (Herberth et al. 2005).

Therefore the present study was undertaken to investigate effects of *L. dahli* venom on liver, heart and kidneys tissues within 72h. Besides, we evaluated efficacy of antivenom to inhibit lethality and neutralizing potency on toxicity effects of *L. dahli* venom within 72h. The findings of the present investigation can be an important step to improve guidelines for an optimal immunotherapeutic treatment of spider envenomation.

## Materials and Methods

### Venom and Antivenom

Crude spider venom and specific antivenom were provided by Department of Venomous Animals and Antivenom Production, Razi Vaccine and Serum Research Institute, Karaj, Iran. In order to obtain the crude venom, *L. dahli* spiders were dissected out, and a pair of glands was collected into ice cold phosphate buffered saline (PBS). The glands were washed in PBS in order to remove possible contaminants, and venom was harvested in PBS by gentle compressing of the glands. The suspension was clarified by centrifugation at 8000rpm, and the venom was stored at −20 °C until use.

### Experimental Protocols

Two groups of New Zealand white rabbits weighing 2±0.5kg (6 animals in each group) were selected. Before the experiment all animals were maintained for at least 3 d, under conditions of controlled light (12h light, 12h dark), temperature (18–22 °C) and humidity (55±5%), with standard diet and water available ad libitum. At first rabbits were anaesthetized with intramuscular injection of 0.5ml ketamine and 0.5ml xylazine in ratio 1:1 respectively. In order to study the hepatotoxicity, myotoxicity and nephrotoxicity of *L. dahli* venom, the first group received only *L. dahli* venom (0.5mg/kg) through subcutaneous route, while the second group 24h after the venom injection, received specific antivenom (2.5ml with neutralization capacity of 500 LD50/ml) by IV route. Seventy-two h after the venom and antivenom injection, experiment ended by scarifying the rabbits for obtaining the heart, liver and kidney tissues throughout the surgery.

### Histological Analysis

After the animals died, the heart, liver and kidneys were removed carefully and immersed in 10% formaldehyde at room temperature and then sectioned transversely into 5μm slices. Specimens were dehydrated in a graded series of alcohol and xylene and embedded in paraffin. Multiple slices (15 fields for each slide) were made and stained by hematoxilin and eosin stains. Sections were viewed and were photographed using a Nikon E200® light microscope (Japan).

This study was approved by Ethics Committee of this institute. Razi Vaccine and Serum Research Institute.

## Results

The signs and symptoms of envenomation appeared within first few hours by redness, mild swelling and muscle cramps at injection site. Twenty-four h after the venom injection difficulty in respiration was observed in most of the animals.

**Fig. 1.**
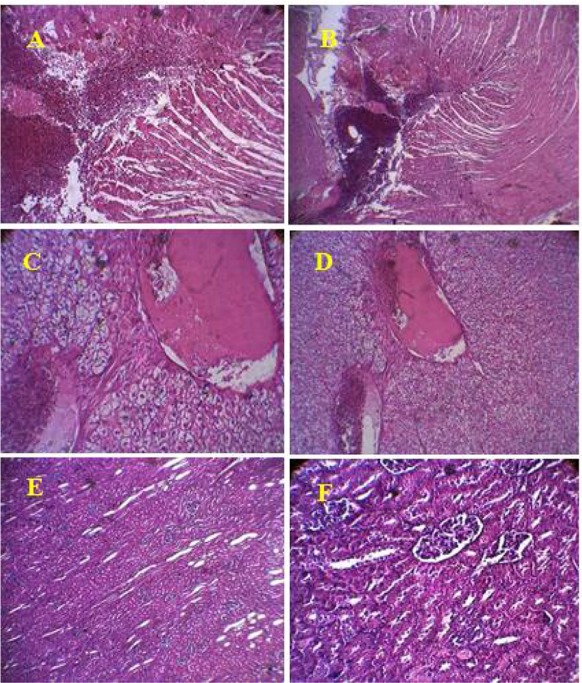
Histopathological changes of heart (A, B), liver (C, D) and kidney (E, F) tissues induced by *Latrodectus dahli* venom (0.5mg/kg, S.C). Within 72h after the venom injection myocytolysis, lymphomononuclear inflammatory infiltrate on the myocardium (A), hemorrhage, myocarditis and myocytolysis (B) are presented. The central vein congestion (C and D) in the liver tissues are presented. Following the venom injection no changes were observed in the kidney tissue (E and F) (Original)

Following 72h after the venom (0.5mg/kg, S.C) and the antivenom (2.5ml, I.V) injection, histological changes were investigated in the rabbit’s heart, liver and kidney tissues in each group. Figure 1 shows the histopathological evaluation of the first group of animals, which venom injection induced complications such as myocytolysis, myocarditis, coagulation necrosis, myocardial edema, hemorrhage and inflammation in the heart tissues (Fig. 1). On the other hand, evaluation of the liver tissues, showed central vein congestion, congested vessels in portal areas, dilated sinusoids and inflammation, however no significant histopathological complications were observed in kidney tissue within 72 h after the venom injection (Fig. 1).

**Fig. 2.**
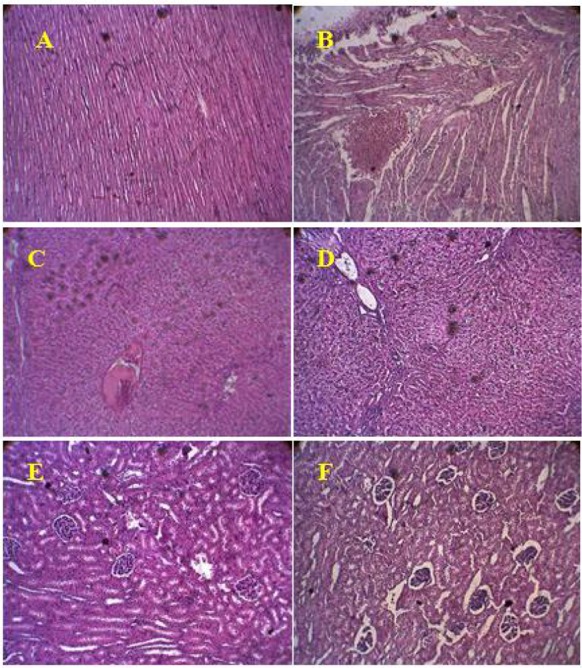
The histopathological changes of heart (A, B), liver (C, D) and kidney (E, F) tissues following the antivenom injection (2.5ml, I.V) 24h after the venom injection. The antivenom prevented severe myocytolysis, edema and hemorrhage in the heart tissue (A), mild myocytolysis and mild hemorrhage are observed in one of the rabbit’s heart (B). The antivenom prevention of the severe central vein congestion and inflammation in the liver are presented (C), mild inflammation were observed in one of the rabbit’s livers in this group (D). No changes were observed in the kidney tissues within 72h after the antivenom injection (E and F) (Original)

In the second group of the animals, immunotherapy prevented severe myocytolysis, edema and hemorrhage in the heart of treated animals. However, in this group, mild myocytolysis and mild hemorrhage in the heart were still observed in the experimented animals (Fig. 2). Antivenom injection also prevented severe central vein congestion and inflammation in the liver. However, mild congestion and mild inflammation were observed in the livers of this group (Fig. 2).

## Discussion

In the present study, the toxic effects of *L. dahli* venom were investigated within 72h on vital organs including the liver, heart and kidney tissues. The findings showed that, the venom injection induced severe cardiovascular complications such as myocytolysis, myocarditis and myocardial hemorrhage in heart. In addition, the venom injection evoked central vein congestion and dilated sinusoids in liver.

The spider genus *Latrodectus*, because of the extreme potency of their neurotoxic compound termed α-latrotoxin, is one of the most notorious spiders in all over the world (Garb et al. 2004). Clinical manifestations of latrodectism, syndrome caused by *Latrodectus*, indicated that the nervous system is the primary target of α –latrotoxin (α-LTX), which follows affecting whole body of organism (Vetter and Isbister 2007). According to our study, it seems that heart is one of the main goals of the venom. Besides, the venom injection induced some histopathological complications such as myocytolysis, myocarditis, coagulation necrosis, myocardial edema and hemorrhage and tissue inflammation. Approximately, in all performed studies on latrodectism, cardiovascular complications have been reported mostly manifested by arrhythmia, tachycardia, bradycardia and ECG changes (Maretic and Stani 1954, Pulignano et al. 1998, Akyildiz et al. 2009). According to the same reports, the changes in ECG waves after black widow spider venom injection were observed clearly, so that ST waves changes and widening of QRS waves indicated myocardial ischemia and myocardial damage, respectively. On the other hand, cardiomyopathy and myocarditis were reported earlier (Akyildiz et al. 2009, Rajabi 2014), detected by reduced height of R and P waves respectively.

Some previous studies (Pulignano et al. 1998, Akyildiz et al. 2009, Rajabi 2014) have reported increasing in cardiovascular enzymatic markers such as CPK, CK-MB and LDH after black widow spider venom injection, which are the indicators of myocardial injury, heart muscle inflammation and arrhythmia. Increasing in foresaid enzymes, are quite compatible to the results of the present study. On the other hand, the histological findings confirmed the incidence of myocarditis, hemorrhage and inflammation in heart tissues after black widow envenomation (Clark et al. 1992, Pulignano et al. 1998, Akyildiz et al. 2009). In addition, occurrence of myocytolysis, and myocardial edema in Marzan and Maretic studies after *Latrodectus* envenomation are another confirmation for the results of the present study (Maretic and Stani 1954, Marzan 1955). The liver and the kidneys because of their extensive blood supply network are as one of the most vulnerable organs to toxin injury (Sitprija and Sitprija 2012). Histological examination performed on the liver tissues showed that within 72h the venom caused central vein congestion, congested vessels in portal areas, dilated sinusoids and inflammation. Marzan and Maretic investigations confirm the pathological effects of the black widow spider venom on the liver tissue. According to their observations, after 30min to 6h of the venom injection, the hepatic cells swollen and gradually massive hyperaemia appears, pericapillary edema after 10 h; necrosis and lobular necrosis were seen respectively after 12 and 24 h of the venom injection (Maretic 1953, Marzan 1955). Following the venom injection, significant increasing in AST and ALT levels are good indicators for cytotoxic effects of the venom on the liver tissue. So that the AST to ALT ratio within 24h after the venom injection was more than 1 (< 1), which from clinical point of view, it is a symptom of impaired liver function (Pulignano et al. 1998, Valikhanfard et al. 2014). These results further confirm our findings in the present study.

The kidney tissues, within 72h after the venom injection, unexpectedly showed no significant histopathological complications. While other histological studies (Maretic 1953, Marzan 1955) have shown that after 10–20h of black widow spider venom injection, degeneration of tubular epithelium with necrosis (within 24 hours) was seen in kidney tissue. Significant increasing in creatinine, bilirubin and urea, as important kidney health parameters, reported within 24h after *Latrodectus* venom injection, represents toxic effects of the venom on kidney function (Maretic 1953, Marzan 1955, Valikhanfard et al. 2014). Although the recent performed studies demonstrate toxic effects of black widow spider venom on kidneys, however we did not observe any significant toxic effects on kidney tissues in our survey.

Other purpose of this study was to evaluate the efficacy of specific antivenom in neutralizing and prevent escalation of the complications on vital organs. According to the obtained results, in the second group of rabbits, the antivenom 24h after the venom injection could protect the heart tissue from severe myocytolysis, edema and hemorrhage. However, mild myocytolysis and mild hemorrhage were observed in some rabbits of this group. In addition, the antivenom prevented histopathological effects on liver tissues, so that except mild congestion and mild inflammation observed in some rabbits; antivenom could protected liver from severe central vein congestion, dilated sinusoids and sever inflammation. Hence it seems that the antivenom injection even 24h after the venom, greatly could prevent incidence and intensification of histopathological complications caused by toxic effects of the venom. However, the mild histopathological effects observed in rabbit tissues may be due to the late (24h after venom injection) injection of antivenom. The obtained results of the antivenom efficacy in the present study corresponded with some studies performed on reducing concentrations in foresaid organs serum enzymes follow intravenous injection of antivenom (Rajabi 2014, Valikhanfard et al. 2014). Based on same studies, 72h after the antivenom injection, significant decreasing was observed in AST, ALT, Urea and Bilirubin, so that the AST to ALT ratio in this period decreased to physiological state, that is less than 1 (< 1). According to the same reports, significant decreasing were observed in CPK, CK-MB and LDH, as predominant heart health markers, also ECG waves returned to its initial state (before venom injection) (Rajabi 2014, Valikhanfard et al. 2014). Black widow spider venom exerts its destructive effects by both direct and indirect pathways. Alpha latrotoxin, the neurotropic venom compound, by interaction with specific receptors on nerve cells, results in releasing of huge amount of catecholamines (Henkel and Sankaranarayanan 1999, Ushkaryov et al. 2008, Silva and Ushkaryov 2010). Hence it seems catecholamines, are the main mediator of the venom effects on organs (Clark et al. 1992). The α-LTX after entering the circulation, through activating L-type calcium channels, which are abundant on vascular smooth muscle cells, results in calcium influx, which leads to vasoconstriction and hypertension (Sitprija and Sitprija 2012).

Catecholamines, by interaction with beta adrenoceptors on cardiovascular system result in activation of L-type calcium channel. Influx of calcium leads to vasoconstriction and hypertension in smooth muscle cells of vascular system, on the heart tissue, activation of L-type calcium channel causes an increase in cytosolic calcium in myocytes, which subsequently induces the cells death (Opie et al. 1985, Van der Heyden et al. 2005). On the other hand, catecholamines by stimulating catecholamine-sensitive lipase in myocardium lead to an increasing in catabolism of free fatty acids. Free fatty acids increasing in myocardium by disrupt the process of oxidative phosphorylation, lead to reduced ATP synthesis, which is other way to induce myocytes death (Opie 1975).

Beside all foresaid mechanisms, according to immunological studies of Herberth in 2005 some latrophilin receptor (α-Latrotoxin independent calcium receptor) genes are expressed in various mammalian tissues, so that the highest expression of latrophilin II can be found in placenta, lungs, liver and mammary glands tissues, respectively. However, some expressions of this gene have also been demonstrated in the heart and kidney tissues (Herberth et al. 2005). Hence, expressions of the latrophilin II, on some of the mammalian tissues, makes the direct effects of the venom expectable, so that latrophilin II by activation of PLC and DAG causes huge released internal calcium from endoplasmic reticulum (Henkel and Sankaranarayanan 1999, Ushkaryov et al. 2008).

It can be predicted that black widow neurotropic venom through interaction with latrophilin receptors expressed on various mammalian organs, induce its cytotoxic effects directly.

## Conclusion

*Latrodectus dahli* venom injection in rabbits, within 72 h, evoked severe cardiovascular complications such as myocytolysis, myocarditis, myocardial edema, hemorrhage and inflammation in heart. In addition, the venom injection induced central vein congestion, congested vessels in portal areas, dilated sinusoids and inflammation in liver. Other results from this study showed specific antivenom injection even 24h after envenomation, greatly could protect the tissues from incidence of histopathological complications induced by the venom.
